# Environmental Enrichment Induces Behavioral Recovery and Enhanced Hippocampal Cell Proliferation in an Antidepressant-Resistant Animal Model for PTSD

**DOI:** 10.1371/journal.pone.0011943

**Published:** 2010-08-05

**Authors:** Hendrikus Hendriksen, Jolanda Prins, Berend Olivier, Ronald S. Oosting

**Affiliations:** Department of Pharmacology, Utrecht Institute for Pharmaceutical Sciences, Utrecht University, Utrecht, The Netherlands; Case Western Reserve University, United States of America

## Abstract

**Background:**

Post traumatic stress disorder (PTSD) can be considered the result of a failure to recover after a traumatic experience. Here we studied possible protective and therapeutic aspects of environmental enrichment (with and without a running wheel) in Sprague Dawley rats exposed to an inescapable foot shock procedure (IFS).

**Methodology/Principal Findings:**

IFS induced long-lasting contextual and non-contextual anxiety, modeling some aspects of PTSD. Even 10 weeks after IFS the rats showed reduced locomotion in an open field. The antidepressants imipramine and escitalopram did not improve anxiogenic behavior following IFS. Also the histone deacetylase (HDAC) inhibitor sodium butyrate did not alleviate the IFS induced immobility. While environmental enrichment (EE) starting two weeks before IFS did not protect the animals from the behavioral effects of the shocks, exposure to EE either immediately after the shock or one week later induced complete recovery three weeks after IFS. In the next set of experiments a running wheel was added to the EE to enable voluntary exercise (EE/VE). This also led to reduced anxiety. Importantly, this behavioral recovery was not due to a loss of memory for the traumatic experience. The behavioral recovery correlated with an increase in cell proliferation in hippocampus, a decrease in the tissue levels of noradrenalin and increased turnover of 5-HT in prefrontal cortex and hippocampus.

**Conclusions/Significance:**

This animal study shows the importance of (physical) exercise in the treatment of psychiatric diseases, including post-traumatic stress disorder and points out the possible role of EE in studying the mechanism of recovery from anxiety disorders.

## Introduction

Exposure to a severe traumatic event will result in a prolonged stress response and anxiety in almost all people. Most people will experience a reduction of such symptoms within one month, however, in about 10–20% of trauma victims the symptoms will not spontaneously recede [1]. The resulting posttraumatic stress disorder (PTSD) is defined as a long-term, maladaptive stress response that involves re-experiencing, avoidance and hyperarousal symptoms (DSM-IV). As such, PTSD can be considered as a failure of recovery after trauma [2]. Treatment of PTSD patients is difficult and currently used pharmacological therapies, mainly serotonergic drugs, suffer from delayed onset, poor efficacy (effect sizes are relative small and many patients do not respond at all) and relapses [3], [4], [5]. Recently, reports about the use of aerobic exercise as a possible (add-on) therapy have emerged [6], [7], [8]. These reports fit in a much larger set of studies in which the beneficial effects of exercise have been studied on psychiatric diseases and cognitive functioning [9], [10], [11].

In animal models, the effects of environmental enrichment and voluntary exercise have been extensively studied. Environmental enrichment gives enhanced possibilities for physical activity and sensory stimulation as compared to standard housing. The availability of a running wheel results in more voluntary (aerobic) exercise, while providing tunnels and shelters and repeated introduction of novel objects leads to more opportunities to experience new sensory information [12], [13]. Environmental enrichment leads to beneficial effects on learning and memory [14], [15], anxiety and depression like behaviors [16], [17] and stimulates recovery after brain lesions [18], [19] or cerebral ischemia [20]. At the cellular level, environmental enrichment enhances neurogenesis in the dentate gyrus [16], [21] and stimulates dendritic branching and spine forming in the CA1 area of the hippocampus [14], [22]. Expression of neurotrophic factors (BDNF, VEGF and VGF) is upregulated by environmental enrichment [16], [23], [24]. Many of the above mentioned changes are also seen after treatment with antidepressants [25], [26], [27].

We investigated the therapeutic potency of environmental enrichment alone and combined with voluntary exercise in an animal model for PTSD. A model for PTSD requires a fear/anxiety inducing event that will lead to a long lasting behavioral effect. Not only contextual conditioned fear should be apparent, but also behavioral changes towards a (novel) context that is not associated with the initial induction of fear must be assessable. Both are core symptoms of PTSD [28], [29], [30]. We applied an inescapable foot shock (IFS) paradigm that induces both long-lasting contextual and non-contextual anxiety [31] and alterations in emotional reactivity measured as a blunted light-enhanced startle [32]. We used reduced locomotion and immobility in an open field test as a measure for anxiety [33], [34]. We show, in agreement with the limited pharmacological treatment possibilities in patients, that imipramine and escitalopram do not improve the behavioral effects induced by IFS. As EE improves depression-like behaviors [16], [17], we expected an improvement of the behavior of shocked rats after EE. Furthermore we expected more resilience to the shocks in animals that were exposed to EE two weeks before IFS since the molecular processes that are held responsible for the antidepressant effect of EE, for example neurogenesis and the expression of neurotrophic factors are also enhanced in “healthy” control animals [16], [23], [24] and thus could form a protective buffer against the IFS. Also, we tested inhibition of HDAC-dependent chromatin remodeling [35], another potential mechanism via which EE may exerts its anxiolytic effect. The usage of two forms of enrichment, with and without a running wheel, shows whether enrichment without extra physical exercise is sufficient for the anxiolytic effect. Finally we determined the effect of IFS and EE on tissue concentrations of monoamines in amygdala, hippocampus and prefrontal cortex.

## Materials and Methods

### Ethics Statement

All animals were handled in strict accordance with good animal practice as defined by the Ethical Committee for Animal Research of Utrecht University, and all animal work was approved by the Ethical Committee for Animal Research of Utrecht University (DEC-ABC).

### Animals

Male Sprague Dawley rats (Harlan, Zeist, The Netherlands) were 8–9 weeks old (between 200 and 220 g) upon arrival. Experimental treatments started 1 week later. Animals were on a 12-hour light/dark cycle.

### Standard housing

Non-enriched cages contained only bedding and one tissue inside a macrolon type IV cage and were cleaned twice a week. Animals were housed three per cage.

### Environmental enrichment

Two types of environmental enrichment (EE) were used: enrichment with and without a running wheel. Enrichment without a running wheel was provided in standard makrolon type IV cages at varying time points relatively to the IFS procedure: two weeks before (EE-continues, N = 12), directly after (EE-after, N = 12) and one week after IFS (EE-delay, N = 12). EE continued until the end of the experiment. Enrichment consisted of a shelter (29×10×12 cm), wooden gnaw sticks (10×2×2 cm), a nesting bag (Datesand Ltd), daily tissues on top of the cage and different sets of tubes (7.5 cm diameter). Three times a week, rats were transferred to a similar but clean cage, provided with a tube set with a different configuration. In these cages also some soiled sawdust bedding (from the old cage) was placed in an open box. Enrichment with running wheel (EE/VE) was provided one week after the IFS procedure until the end of the experimentint in a larger cage (57×45×35 cm), the rest of the enrichment was similar as described above.

### Inescapable footshock procedure (IFS)

The rat was placed in a light/dark box that consisted of a brightly (230 lux) lit (white) compartment (23×19×35 cm) and a dark (black) compartment (29×23×35 cm) with stainless steel rods, connected by a sliding door. Once the animal entered the dark compartment the door was closed and 10 shocks (1 mA) of 6 seconds duration, randomly divided over a 15-minute period were given. All rats from the same cage were shocked at the same time and then placed back together in their home cage. Control animals were left undisturbed.

### Open field test

The open field arena consisted of a dark gray box (72×72×45 cm with a black floor). Illumination was ±20 lux at floor level. During the test a 80–85 dB background white noise was presented. Each animal was placed in the center of the open field before recording started. After 5 min they were returned to their home cage or the so-called stress of sudden silence was applied (see below). Behavior was recorded and later analyzed using Noldus EthoVision (Noldus Information Technology). After each session the floor of the open field arena was cleaned with 70% ethanol.

### Stress of sudden silence (SOS)

After the first 5 minute in the open field arena with background noise the sound was turned off. Freezing behavior (by a skilled observer, who was unknown of the treatment the animal had received) and locomotion during the next 5 minutes was recorded.

### Light/dark box

The rats were placed in the lit compartment of the box that was used for the IFS procedure for 30 seconds and then the sliding door was opened. During the next 5 minutes, we scored 1) latency to enter the dark compartment, 2) the amount of time spent in each compartment and 3) the number of times the animals changes from compartment. The light/dark box experiments were performed three weeks after IFS.

### BrdU staining

Bromodexoyuridine (BrdU, Sigma) (20 mg/ml dissolved in 7 mM NaOH and 0.15 M NaCl) was injected (i.p.) twice at a dose of 150 mg/kg. BrdU injections were given either immediately after the IFS procedure with a 20 hours interval between the two injections, or four weeks after the IFS, 24 hours before sacrificing the animals, with a 2 hours interval between the two injections. Rat brains were fixed *in situ* using 4% paraformaldehyde in phosphate-buffered saline (PBS), and finally stored at −20°C immerged in 30% glucose in PBS for cryopreservation. 40 µm brain slices were cut at −18°C and stored until use in PBS at 4°C. Slices were immunolabelled with anti-BrdU (Roche Diagnostics, diluted 1∶3000) as described by Heine et al [36].

### Quantification of BrdU labled cells

In every 5^th^ section throughout the hippocampus the number of BdrU-positive cells was counted in the hilus, subgranular zone (SGZ), Granule cell layer(GCL) and the molecular layer, over the entire rostro-caudal extent of the hippocampus. The number of counted cells was multiplied by 5 to obtain the total number of positive cells in the dentate gyrus.

### Drugs

Imipramine-HCL (Sigma) was administered intraperitoneally (i.p.) at 20 mg/kg in a dose volume of 2 ml/kg for 14 consecutive days starting one week after the foot shock procedure. Ecitalopram-HCL was given orally for 14 days at a dose of 10 mg/kg (5 ml/kg) starting one day after the foot shock procedure. Sodium butyrate (Sigma Aldrich, Zwijndrecht, The Netherlands) was given (i.p.) at a dose of 300 mg/kg (2 ml/kg) for 14 days starting one week after the IFS procedure. The drug doses were based on literature (imipramine [37], escitalopram [37], sodium butyrate [38]) To prevent direct (e.g. sedating) effects drug administration was ended 24 hours before the open field test. To reduce the number of animals needed, we did not include an imipramine no IFS group. In previous experiments we have repeatedly shown that chronic imipramine treatment had no effect on locomotion of control animals [37].

### HPLC analysis of monoamines

Tissue samples (amygdala, prefrontal cortex and hippocampus) were weighed and stored at −80°C until use. The methods for sample preparation and HPLC analysis were taken from [39].

### Statistical analysis

The data obtained from the open-field and SOS tests were analyzed by ANOVA, followed by posthoc analyses with Bonferonni corrections for multiple comparisons. When data violated the assumption of sphericity, Greenhouse-Geisser correction was applied. Non-parametric statistics (Kruskal-Wallis and Mann-Whitney test) were used to analyse the light/dark box behavior. For all statistical analyses, *p*-values <0.05 were regarded significant. Normal distribution of data was analyzed using the Kolmogorov-Smirnov test. All statistical analyses were carried out with SPSS v16.0.

## Results

### Anxiogenic effects of Inescapable Foot Shock (IFS) are long lasting

**Figure 1:**
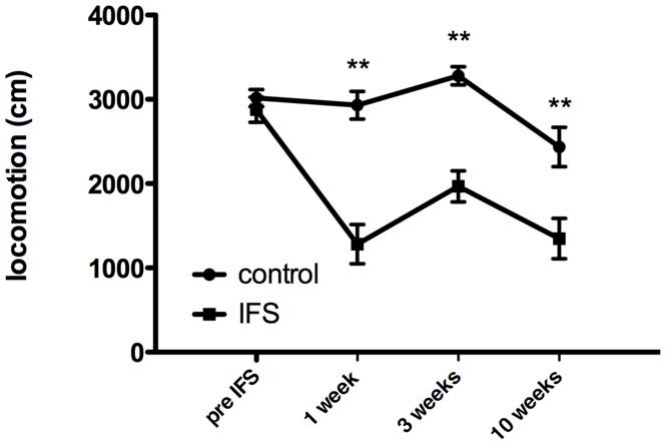
Longitudinal effects of IFS on open field locomotion. Anxiolytic effects of the inescapable foot shock procedure are long lasting. The distance moved in the open field arena during a 5 minute test period is shown. (repeated measure ANOVA, p<0.001). Values represent mean ± SEM. Group sizes: non-shock n = 10, shock n = 10, ** =  post hoc analysis p<0.01.

Repeated measures ANOVA showed a significant interaction (F(3,54) = 6.093, p<0.001) between the IFS procedure and the distance moved over time in the open field (Figure 1). Even ten weeks after the inescapable foots shocks (IFS) animals showed a significantly lowered locomotion (Bonferroni, p = 0.004).

**Figure 2:**
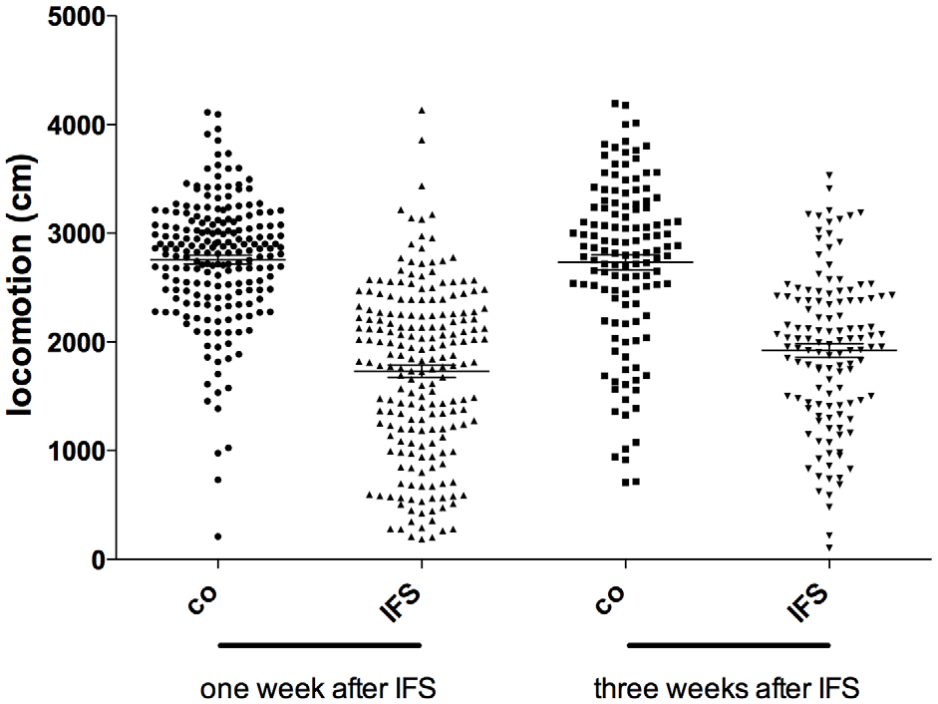
Distance moved in open field per individulal animal. Distance moved in the open field arena one week and three weeks after the IFS procedure. Group sizes were: one week post shock, control n = 207, shock: n = 207, Three weeks post shock, control n = 127, shock: n = 127.

To investigate whether a population of Sprague Dawley rats consisted of subpopulations of sensitive and resilient animals, we plotted for each control or IFS exposed animal that we used during the last two years the distance moved in the open field arena one (n = 204) and three weeks (n = 127) after the IFS procedure (Figure 2). Visual inspection of the data one week after the shock does not reveal a subpopulation of more susceptible animals, although this large sample of animals does not have a normal distribution (Kolmogorov-Smirnov, KS = 0.078, p = 0,004). However, three weeks after the IFS the “distance moved” parameter was normally distributed (Kolmogorov-Smirnov, KS = 0.063, p = 0.20) suggesting that all rats had more or less similar sensitivity to the long-lasting effects of the IFS procedure.

### Environmental enrichment has anxiolytic effects on IFS rats

In order to investigate possible protective and therapeutic aspects of environmental enrichment (EE) on IFS-induced anxiety, we introduced EE (without running wheel) to the animals at different time points relative to the IFS procedure. EE started either two weeks before (EE-continues), immediately after (EE-after) or 1 week after the IFS procedure (EE-delayed). For each group the EE exposure continued until the end of the experiment. We also included standard-housed control groups (SH).

**Figure 3:**
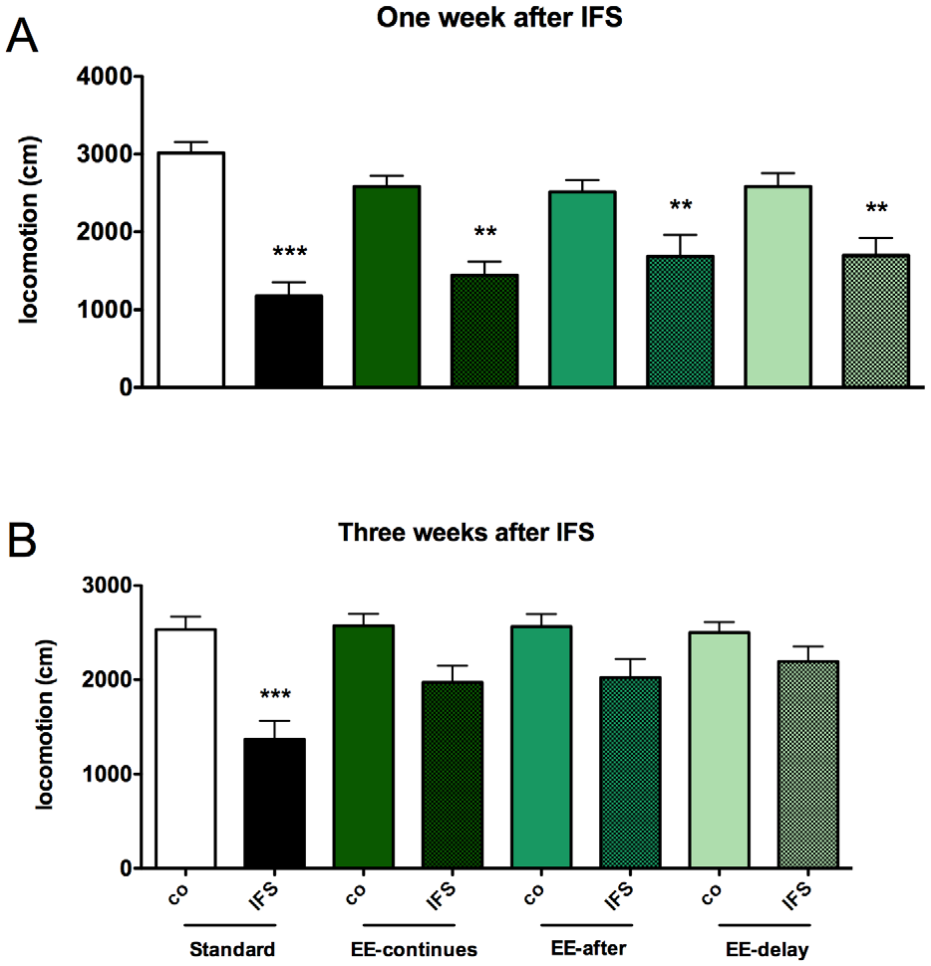
Effects of environmental enrichment on locomotion in the openfield. (**A**) Environmental enrichment (EE) had no significant effect on locomotion in the open field one week after IFS. (**B**) Three weeks after the IFS EE normalizes open field behavior. Environmental enrichment started either 2 weeks before (EE-continues), immediately after (EE-after) or 1 week after (EE-delayed) the IFS procedure and continued until the end of the experiment. Values represent mean ± SEM. (*p<0.05, **p<0.01, ***p< 0.001). Group sizes: non-shock n = 12, shock n = 11

One week after the IFS procedure, all IFS- groups were significantly different from their non-shocked control group as analyzed by two-way ANOVA, F(1, 88) = 59.589, p<0.001). (see Figure 3A); no effect of housing was found (F(2, 88) = 0.237, p = 0.790). Three weeks after IFS (Figure 3B), there was a significant effect of housing condition on the shocked animals (F(1,90) = 8.213, p = 0.005). The locomotion of standard housed shocked animals was still significantly reduced as compared to the non-shocked control group (Bonferonni p< 0.001), while all other shocked, enriched, groups did no longer differ from their controls as shown by Bonferonni's multiple comparison test.

### EE-induced cell proliferation in hippocampus correlates with behavioral recovery

**Figure 4:**
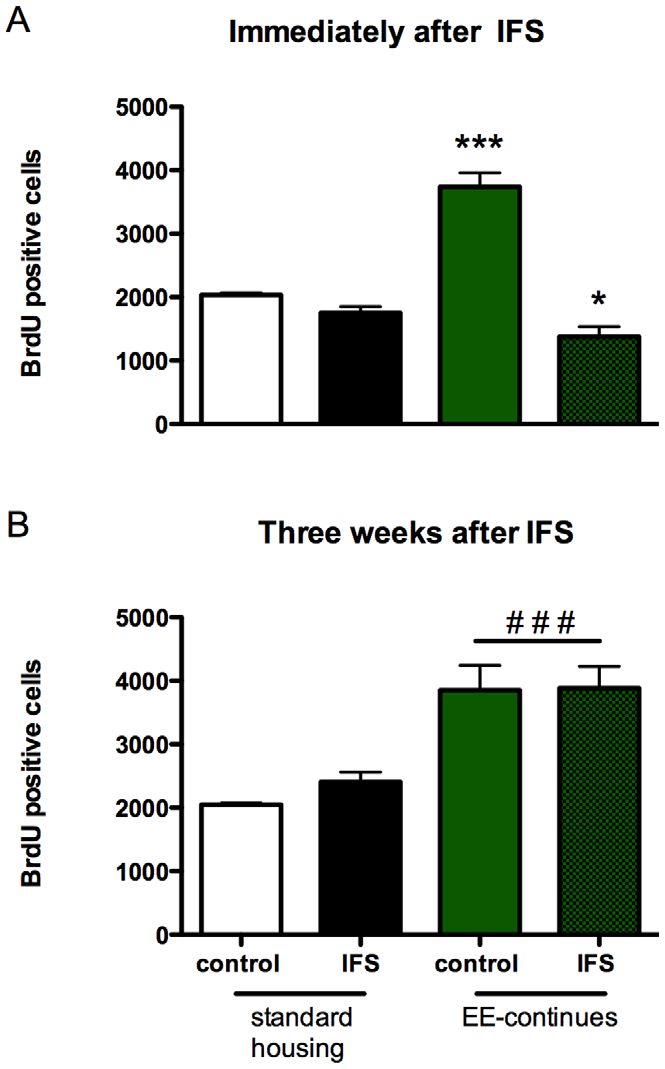
Effects of IFS and environmental enrichment on cell proliferation/survival. Environmental enrichment started two weeks before the IFS procedure (EE-continues). (**A**) BrdU was injected immediately after the IFS procedure. In this way proliferation and survival during the three weeks post IFS was measured. (**B**) To quantify proliferation three weeks after the IFS procedure, BrdU was injected 21 days after the IFS and 24 hrs later the animals were sacrificied. BrdU incorporation was quantified by immunocytochemistry. Values represent mean ± SEM. Group sizes: non-shock n = 6, shock n = 6. * =  post-hoc analysis significanty different from its control group p<0.05, ***  = p<0.001. ### =  ANOVA significantly different effect of housing p<0.001.

The effect of the IFS procedure on cell proliferation in the dentate gyrus of the hippocampus was studied in the EE-continues group. Animals were injected with BrdU either immediately after the IFS procedure (in this way proliferation and survival during the three weeks post IFS is measured, Figure 4A) or 3 weeks later, 24 hrs before sacrificing the rats (Figure 4B).

As is clear from Figure 4A, EE almost doubled the number (Bonferroni p<0.001) of proliferating/surviving cells in the dentate gyrus compared to standard housed (SH) animals. Importantly, immediately after the IFS procedure, the number of proliferating cells in the EE animals decreased to levels even lower than that of SH animals (Bonferroni p = 0.03). Two-way ANOVA revealed a significant effect of housing (F(1,18) = 20.91, p<0.001) and significant interaction (F(1,18) = 50.83, p<0.001) between housing and shock. In SH animals, IFS did not lead to a further reduction in cell proliferation/survival. Three weeks after the IFS procedure, cell proliferation in the dentate gyrus was not longer affected by the IFS procedure: The number of proliferating cells in the EE-continues group was comparable to the number of proliferating cells in the non-shocked EE-continues group and was almost twice as high as in the SH groups (F(1,18) = 36.27, p<0.001).

### Anxiolytic effects of environmental enrichment combined with voluntary exercise

**Figure 5:**
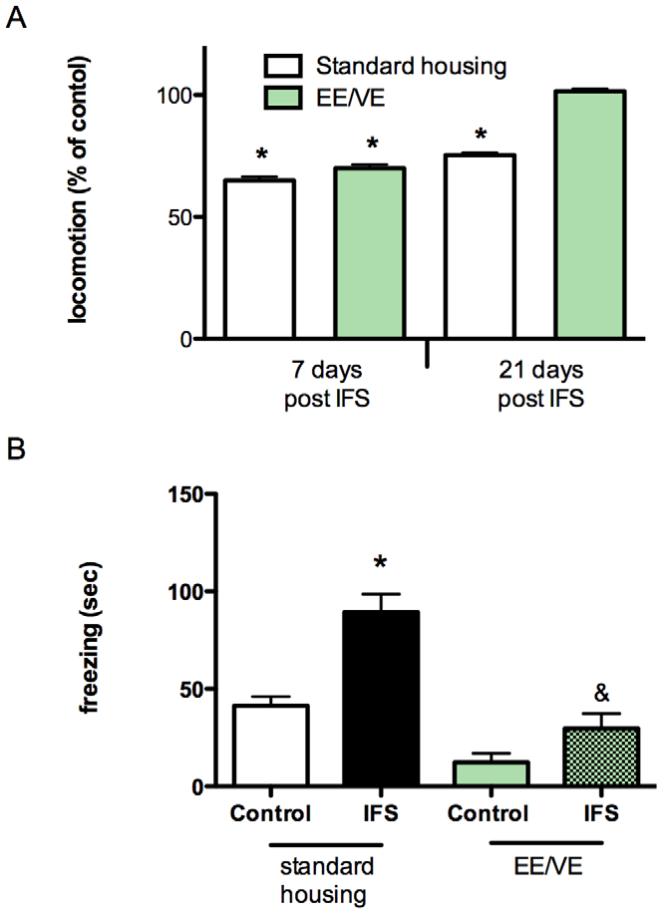
Environmental enrichment combined with voluntary exercise normalizes open field behavior three weeks after IFS. EE/VE started 1 week after the IFS procedure. (**A**). A percentage relative to non-shocked animals of the distance moved in the open field arena during a 5 minute test period is shown. (**B**) EE/VE normalizes the freezing behavior of IFS-exposed animals. *p<0.05 compared to non-shock control group. ^&^p<0.05, compared to the IFS group under standard housing conditions. Values represent mean ± SEM. Group sizes: non-shock n = 12, shock n = 12.

In a next series of experiments, partly done to duplicate and extend our previous findings, we combined environmental enrichment with voluntary exercise (EE/VE). Animals were subjected to the IFS procedure and one week later exposed to environmental enrichment with a running wheel. Two-way ANOVA revealed significant interaction between EE/VE and the locomotion of shocked animals (F(1,44) = 10.068, p = 0.003). Complete recovery in open field behavior of the shocked animals was observed after EE/VE (see Figure 5A). As an additional parameter we measured, at the three week time point, the response of the animals in the so-called SOS test (see Figure 5B). Standard housed IFS- animals showed an increase in freezing behavior in the five minutes after turning off the background noise (Bonferroni p<0.001). IFS-animals housed under EE/VE conditions, however, showed no differences in freezing behavior compared to the non-shocked group (p = 0.470). Two-way ANOVA shows a significant interaction between housing and shock (F(1,44) = 5.00, p = 0.03)

### Effects of environmental enrichment/voluntary exercise (EE/VE) on behavior in the light/dark-box

**Figure 6:**
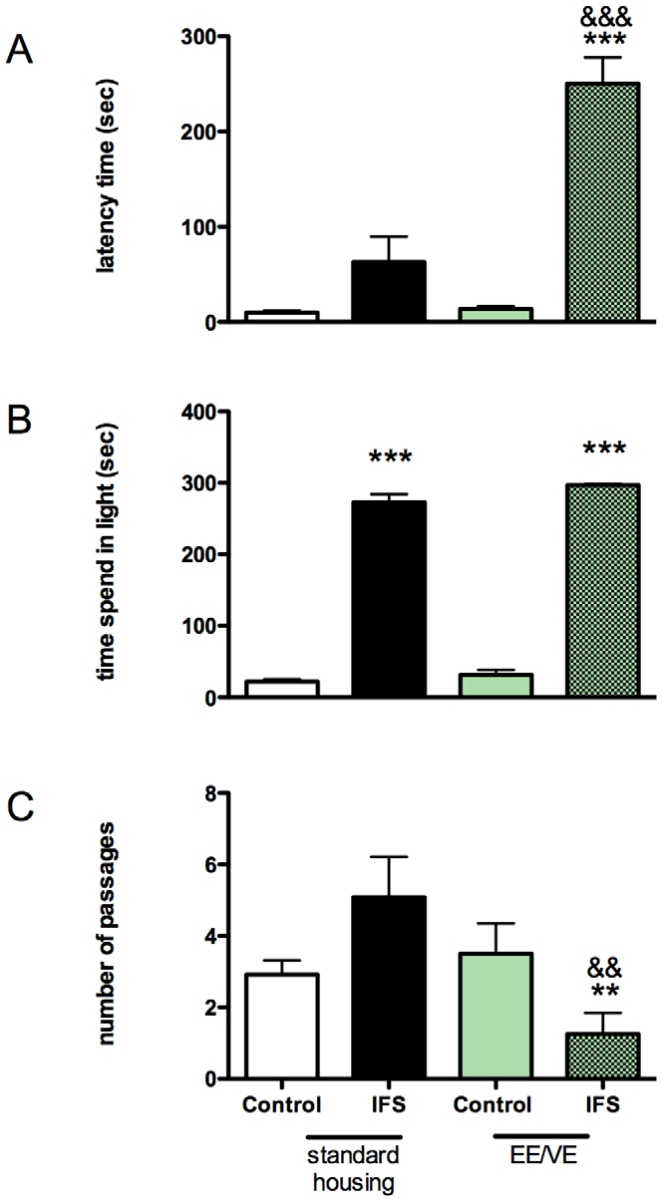
Behavior in the light/dark box. The EE/VE induced normalization of the open field behavior of the IFS-exposed animals is not caused by loss of memory to the traumatic experience. Animals were placed in the lit compartment of the light/dark box (door open) that was used for the IFS procedure and (**A**) the latency to enter the dark compartment, (**B**) the total time spent in the lit compartment was quantified and (**C**) the number of passages from the lit into the dark compartment or vice versa during a five minute trial * =  Mann Whitney test significantly different from control group; p< 0.05, ** = p<0.01, *** = p<0.001, &  =  significantly different from IFS standard housed group. Values represent mean ± SEM. Group sizes: non-shock n = 12, shock n = 12.

We tested the animals in the same light/dark-box that was used for the IFS procedure (Figure 6). The animals were placed in the light compartment and were allowed to move to the dark shock compartment and vice versa. IFS animals housed under EE/VE conditions entered the dark compartment with a latency time of almost 4 times longer than standard housed IFS animals (Mann-Whitney U = 2.00, z = −4.1, p<0.001). Most enriched IFS animals did not enter the dark compartment at all. IFS animals housed under EE/VE conditions changed compartments less frequent than standard housed IFS animals (Mann-Whitney U = 23.00, z = −2.89, p<0.004). Both IFS standard housed animals and IFS animals housed under EE/VE conditions spend significantly more time in the lit compartment, U<0.001, z = −4.16, p<0.001 and U<0.001, z = −4.24, p<0.001 respectively.

### Antidepressants or HDAC inhibition did not stimulate behavioral recovery of IFS rats

Both chronic administration of the tricyclic antidepressant imipramine (10 mg/kg) and the selective 5-HT transporter-blocker escitalopram (10 mg/kg) were unable to restore open field behavior of the IFS rats (see Table 1) The effect of IFS was still present after administration of escitalopram (F(1,55) = 31,18, p<0.001) and there was no interaction between the effect of IFS and the drug effect (F(1,55) = 0.026, p = 0.087). There was a significant difference between non-shocked control escitalopram treated group and the IFS group treated with escitalopram (Bonferroni p = 0.002). The IFS group treated with imipramine was still significantly different from the non-shocked control animals (Bonferroni p = 0.004) but was not significantly different from the saline treated IFS group (Bonferroni p = 0.109).

**Table 1:** Locomotion in the open field after chronic (14 days) pharmacological treatment.

Escitalopram	distance moved
Control saline	3136±135 cm
Control escitalopram	2924±189 cm
IFS saline	2063±185 cm**
IFS escitalopram	1910±228 cm**
**Imipramine**	
Control	3003±134 cm
IFS	1904±261 cm**
IFS Imipramine	1386±241 cm**
**Sodium butyrate**	
Control saline	3333±164 cm
Control Sodium butyrate	3128±160 cm
IFS saline	2511±160 cm**
IFS Sodium Butyrate	2007±143 cm***

Values represent mean ± SEM. Group sizes: n = 12.

** =  p<0.01,

*** =  P<0.001 compared to non-shock control group.

To investigate whether inhibition of HDAC-dependent chromatin remodeling could mimic the anxiolytic effect of enrichment, we treated IFS rats housed under standard non-enriched conditions, starting one week after the shock procedure, for two weeks with the HDAC inhibitor sodium butyrate. There was still a shock effect F(1,44) = 42,68, p<0.001. Non-shock controls and IFS animals were still significantly different (Bonferroni p<0.001) after treatment with sodium butyrate. There was no significant interaction between shock and drug effect (F(1,44) = 1.011, p = 0.32. This treatment did not affect recovery (see Table 1)

### Changes in monoamines levels in hippocampus, amygdala and prefrontal cortex following IFS and EE/VE

#### Noradrenalin

**Figure 7:**
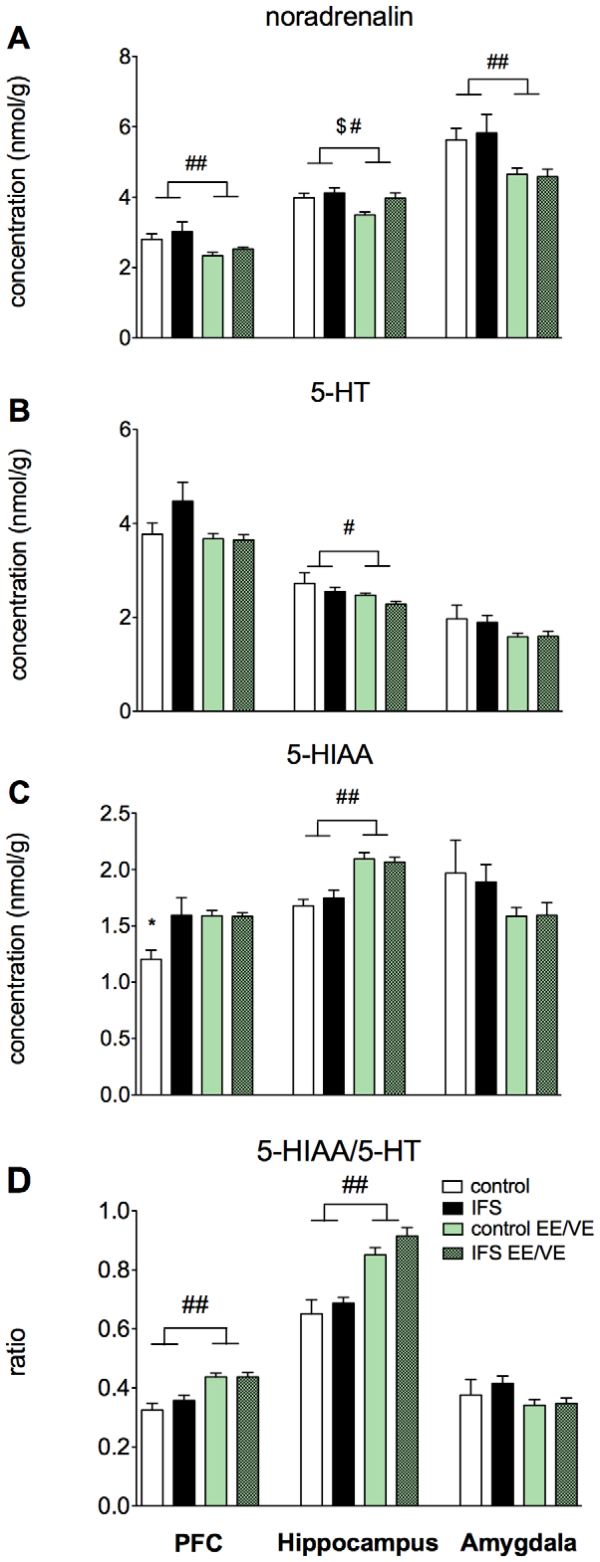
Monoamines levels in prefrontal cortex, hippocampus and amygdala following IFS and EE/VE. Levels of noradrenalin (**A**), 5-hydroxytryptamine (5-HT) (**B**) and 5-hydroxyindole-3-acetic acid (5-HIAA) (**C**) in the prefrontal cortex (PFC), hippocampus and the amygdala. The ratio of 5-HIAA and 5-HT (**D**) is indicative for the 5-HT turnover. # =  effect of housing p<0.05, ## =  effect of housing p<0.01, $ =  effect of shock p<0.05, *  =  post hoc p<0.05. Values represent mean ± SEM. Group sizes: n = 10–12.

Concentrations of noradrenalin, 5-hydroxytryptamine (5-HT) and 5-hydroxyindole-3-acetic acid (5-HIIA) were measured in the prefrontal cortex (PFC), hippocampus and the amygdala three weeks after the IFS procedure of animals housed under standard or EE/VE conditions. Noradrenalin levels in the prefrontal cortex (Figure 7A) were significantly (F(1,44) = 8.18, p = 0.006) lower in the EE/VE animals however, there was no effect of IFS on noradrenalin levels in the PFC (F(1,44) = 1.53, p = 0.222). In the hippocampus noradrenalin levels were also lower in enriched animals (Two-way ANOVA F(3,88) = 5.99, p = 0.018). The IFS procedure increased the noradrenalin levels (F(1,44) = 5.37, p = 0.025). However, there was no interaction (F(1,44) = 1.674, p = 0.202) between the effect of the enrichment and the IFS procedure on noradrenalin levels. Levels of noradrenalin in the amygdala were significantly lower in animals housed under enriched conditions (F(1,40) = 10.3 p = 0.003). There was no interaction with the schock condition.

#### 5-HT/5-HIAA

In PFC, 5-HT levels were unaltered by IFS and EE/VE. However, the levels of 5-HIAA, the major metabolite of 5-HT, were significantly higher in the three experimental groups (IFS-standard housed, non-shocked-EE and IFS-EE animals) compared to non-shocked standard housed animals (see Figure 7B–D). EE animals showed a higher 5-HIAA/5-HT ratio in the PFC, indicative of an increased 5-HT turnover (F(1,44) = 29.551 p<0.001). In hippocampus, levels of 5-HT were significantly lower (F(1,44) = 4.163, p = 0.047) in both enriched groups. The levels of 5-HIAA in the hippocampus were significantly higher in enriched animals (F41.621, p<0.001). The ratio 5-HIAA/5-HT was significantly higher (F(1,44) = 43.947, p<0.001) for enriched animals. In the amygdala levels of both 5-HT and 5-HIAA were unaltered.

## Discussion

Pharmacological treatment for post-traumatic stress disorder (PTSD) is not very effective [3], [4], [5], [40]. Here we provide in an animal model evidence for a therapeutic effect of environmental enrichment and exercise in the treatment of PTSD. Animals housed under enriched conditions recovered much faster from the effects of an inescapable foot shock procedure than animals housed under standard conditions. Such a fastened recovery was not found following chronic administration of imipramine or escitalopram. Both antidepressants are routinely used for the treatment of PTSD, although their efficacy is dubious [41]. Importantly, EE given two-weeks before the IFS does not protect the rats from the initial effects of the foot shock procedure. We used two types of cage enrichment; enrichment with and without a running wheel. We did not measure freezing behavior (in the SOS test) of the animals that were exposed to the EE without a running wheel. Therefore, we can't perform a direct comparison of the anxiolytic effect of the two types of enrichment. The results suggest however, that the anxiolytic effect is not due to exercise per se.

In the IFS procedure rats are shocked repeatedly during 6 seconds divided over a 15 minute period. The behavioral effects were long-lasting. Even after 10 weeks, the effects of the IFS procedure were evident (see Figure 1). IFS-treated animals show reduced mobility in an open field and had an exaggerated freezing response in the SOS test (Figure 5B). An animal model for PTSD should show not only contextual conditioned fear, but should also show behavioral changes towards a (novel) context that is not associated with the initial induction of the fear [29], [30]. The IFS rats clearly show contextual fear (Figure 6). Importantly, this fear memory was not affected by EE/VE. This is logic from an evolutionary standpoint, since a dangerous environment should be avoided whenever possible. The conditions/context during the open field and SOS tests were very different from the conditions during the IFS procedure and thus, at least in this aspect, the IFS-induction model meets the criteria for a PTSD model.

In humans, “only” 10–20% of individuals that are exposed to a severe trauma develop PTSD [1], indicating that the majority is resilient. This seems in contrast with our observation that the majority of our animals show long-lasting signs of trauma related anxiety (Figure 2). However, epidemiological research reveals a robust dose-response relationship between the severity of the trauma and the development of PTSD [1], [42]. Therefore it is likely that our IFS procedure is such a severe stressor that it induces a post traumatic stress behavior in the majority of exposed animals.

One of our findings was that rats when housed under EE conditions are not protected against the initial effects of the IFS procedure (Figure 3). We cannot exclude the possibility that two weeks of EE prior to IFS is too short to protect the animal from the behavioral effects of the trauma, although this is unlikely since two weeks of EE after the IFS procedure is enough for recovery. In mice, it has been shown that EE/VE protects against the behavioral effects of mild stressors, such as occur during the elevated plus maze or the open field test [16], [43]. Here we show (Figure 5B) that EE/VE makes the non-shocked control rats less reactive in the SOS test, compared to standard housed rats. And thus EE/VE probably protects against the (acute) effects of relatively mild stressors, but does not protect against the effects of more severe stressors. This conclusion is supported by data from rats who were housed for four weeks with a running wheel and then exposed to an inescapable foot shock procedure [44]. In this experiment ISF rats were less active during the first week after the shock compared to the non-shocked controls. At later time points, the activity of shocked rats recovered to control activity levels. Although running wheel activity is probably not directly related to anxiety, this parameter may be indicative for the general well being of the animal.

A potential mechanism why rats housed under EE condition recover faster from the effects of severe trauma is that they learn to cope with (mild) stressors, and thus also with the stress during the open field paradigm. EE exposed animals received every two days a clean cage with a new configuration of tubes, new bedding material, etc. From telemetry studies performed in our lab, we know that receiving a new cage is stressful for a rat, both its body temperature and heart rate increase [45]. The increased ability of the EE/VE animals to cope with stress may also be deduced from the behavior of these animals during the dark/light box experiments (Figure 6). Independent of housing conditions, shocked animals spend much more time in the lit compartment and thus develop an aversive association with the dark compartment. However, the standard housed IFS animals exhibit a shorter latency to leave the lit compartment and to enter the dark compartment where they were shocked. Almost all animals of the enriched IFS group, however, remain in the light compartment until termination of the test. Moreover, standard housed IFS animals change compartments 5 times on average but still show a substantial aversion for the dark compartment, suggesting an inability in the standard housed IFS animals to cope with the stressful situations in the lit and in the dark compartment. Together with our findings that EE reduce freezing behavior in non-shocked animals after the mild stressor in the SOS test, these results suggest changes in coping style as underlying mechanism of action of environmental enrichment.

An important question is which cellular and molecular changes underlie the behavioral recovery promoting effects of EE? The increase in cell proliferation observed after two weeks of EE, before the IFS, is completely back to standard housed control levels directly after IFS (Figure 4A). This correlates with the attenuated locomotion one week after IFS in the open field. Continued exposure to EE after IFS for 3 weeks enhances cell proliferation almost 2-fold and normalizes the locomotion in the open field. This might suggest that cell proliferation plays a role in EE facilitated recovery after stress, as also shown by Schloesser et al [46]. On the other hand, it has been shown that anxiolytic effects of EE are independent of hippocampal neurogenesis in experiments in which neurogenesis in the hippocampus was blocked by X-radiation [47]. This, together with our finding that chronic antidepressants treatment, which also induces neurogenesis in the hippocampus [48], does not improve the behavior of the IFS-rats, might indicate that additional mechanisms are responsible for recovery after a severe stressor. However, we cannot exclude the possibility that the degree of cell proliferation is important in inducing an anxiolytic effect. The levels of cell proliferation due to wheel running are much higher than the antidepressant induced cell proliferation [49]. Although our results cannot elucidate the question whether cell proliferation is obligatory for the anxiolytic effect of EE, the above-mentioned literature and our findings suggest that EE-induced effects on neurogenesis and the anxiolytic properties of EE have, at least partly, common underlying mechanisms. One such mechanism may be increased levels of neurotrophic factors, like BDNF and VEGF. Recently, we showed that EE/VE in Sprague-Dawley rats increases the mRNA levels of VEGF in the hippocampus, without having an effect on the mRNA levels of BDNF and VGF (Hendriksen et al, unpublished results). These neurotrophic factors probably affect many neuronal processes/pathways, including dendritic branching and spine number and more importantly the action of these neurotrophic factors is probably not limited to the hippocampuss [50], [51] (although as we found previously (unpublished results), EE/VE does not affect the levels of VEGF in prefrontal cortex).

We also studied the effects of histone deacetylase (HDAC) inhibition. The idea behind these experiments was primarily based on the work published by Fischer et al [18], [35] and Tsankova et al [52] who showed in an animal model for neurodegeneration that HDAC inhibition (using sodium butyrate) results in comparable neuro-restorative effects as induced by EE. Their hypothesis is that changes in chromatin remodeling as induced either by EE or sodium butyrate underlies the required rewiring of the remaining neurons. Tsankova et al showed that HDAC inhibition is an essential step in the behavioral effects of imipramine in the social defeat depression model. In our IFS model, HDAC inhibition did not have any anxiolytic effect.

Involvement of the hippocampus, amygdala and prefrontal cortex, in the manifestation of PTSD is presented abundantly in literature [53], [54]. Therefore, we have investigated in these areas whether EE/VE affects concentrations of the major monoamines and how possible changes correlate with the EE-induced behavioral recovery. Since 5-HT levels are enhanced by both imipramine and escitalopram, which both failed to improve the IFS induced behavior, we consider it implausible that the changes of basal levels of 5-HT in the hippocampus or the augmented 5-HIAA/5-HT ratio are primary responsible for the anxiolytic effect of EE. However it is likely that the enhanced turnover of 5-HT contributes to the positive effect of enrichment. In all three examined brain areas basal noradrenalin levels are lower in enriched animals (see Figure 7A). In particular a reduced activation of beta-adrenergic receptors could be responsible for the effects of EE/VE, since propranolol, a centrally acting beta-adrenergic receptor antagonist reduces fear both in humans and in experimental animals [55]. In the PFC lower levels of noradrenalin might add to its capacity to inhibit hyperactivity of the amygdala [56]. The precise role of noradrenalin in the anxiolytic effect of environmental enrichment is not yet clear however, our results solicits for further research into this putative key player.

In conclusion, we showed in an animal model of PTSD the beneficial effects of environmental enrichment/increased exercise on the speed of behavioral recovery following severe trauma. The challenge will now be to identify those processes/pathways that underlie the EE-induced behavioral recovery. It seems likely that those processes share, at least partly, common underlying mechanisms with enhanced cell proliferation.
